# APE1 localizes to chloroplast stromules and interacts with ATI1 in Arabidopsis

**DOI:** 10.1080/15592324.2025.2511830

**Published:** 2025-05-30

**Authors:** Zihan Ge, Yunhe Jing, Jiaoyu Zhu, Li-En Yang, Shan Lu, Yinyin Deng

**Affiliations:** a School of Life Sciences, Nanjing University, Nanjing, China; b Jiangsu Marine Fisheries Research Institute, Nantong, China

**Keywords:** *Arabidopsis thaliana*, ACCLIMATION OF PHOTOSYNTHESIS TO ENVIRONMENT1 (APE1), ATG8-INTERACTING PROTEIN1 (ATI1), protein–protein interaction, stromule

## Abstract

Chloroplast stromules are dynamic tubular extensions implicated in inter-organelle communication and stress responses in plants. However, the molecular mechanisms underlying stromule-mediated processes remain largely unexplored. In this study, we investigated the localization and function of ACCLIMATION OF PHOTOSYNTHESIS TO ENVIRONMENT1 (APE1) and its interaction with the autophagy-related protein ATG8-INTERACTING PROTEIN1 (ATI1) in *Arabidopsis thaliana*. Expression analysis revealed that *APE1* is predominantly expressed in rosette leaves. Using yeast two-hybrid and luciferase complementation assays, we confirmed a direct interaction between APE1 and ATI1. Confocal microscopy demonstrated that APE1 localizes both within chloroplasts and stromules, co-localizing with stromule markers, while sub-organelle fractionation indicated APE1 enrichment in the thylakoid and inner envelope membranes. Our findings suggest that APE1, through its interaction with ATI1, may play a crucial role in stromule-mediated signaling and the selective autophagic degradation of chloroplast proteins during stress acclimation. This work provides new insights into the functional dynamics of stromules and highlights the importance of the APE1–ATI1 interaction in chloroplast-nucleus communication under environmental stress.

## Introduction

Plants are constantly exposed to a wide array of environmental stresses such as high light, drought, salinity, and pathogen/herbivore attacks. To survive and thrive under these challenging conditions, plants have evolved diverse and highly controlled mechanisms to avoid, relieve, or repair the damage. One of the central cellular machineries is autophagy – a highly conserved cellular process that enables them to recycle cellular and organelle components efficiently.^1,2^ Autophagy-related genes (*ATG*s) play a vital role in orchestrating this process, ensuring proper stress responses and overall plant health.^3,4^

Chloroplasts are the semi-autonomous organelles for plants, with critical functions such as photosynthesis and protein translation. Damages to chloroplasts not only affect photosynthesis but would also ultimately impede plant growth.^5,6^ Repairing or eliminating damaged chloroplasts and their proteins is a foundation for preserving optimal chloroplast functions and preventing further cellular damage.^7,8^ ATG8 and ATG8-interacting proteins (ATIs) stand out as key components for the turnover of chloroplasts and their proteins achieved through autophagy, with ATG8 governing the selective recognition and ATI1 transporting the chloroplast cargo to vacuoles for degradation.

Previous studies indicated that ATI1 and ATI2 are present on the surface of plastids, in structures known as ATI-PS bodies and stromules, both of which are budding and detaching from plastids under stressed conditions to contribute the tolerance of plants.^4,9,10^ These bodies harbor chloroplast proteins destined for degradation in the cytosol or lytic compartments (the vacuole in plants). In addition to ATI-PS bodies, ATIs are also found in stromules – narrow, tubular structures composed of stroma and surrounded by the plastid envelope membrane. It was interesting that ATI1 was reported to interact with ACCLIMATION OF PHOTOSYNTHESIS TO ENVIRONMENT1 (APE1, AT5G38660).^10^ Identified through chlorophyll (Chl) fluorescence-based screening, *ape1* is among a group of *Arabidopsis thaliana* mutants defective in acclimation of photosynthesis to the light environment.^11^ Sharing no significant sequence similarities with other proteins, APE1 is indispensable for the proper adjustment of PSII activity and the maintenance of a balanced Chl *a*/*b* ratio, which together suggest a contribution to the plant’s ability to mitigate photodamage during high light stress, albeit the underlying molecular mechanism is still unknown.^11^ Therefore, the ATI1–APE1 interaction may bridge two fundamental processes in plant biology, autophagy and photosynthetic acclimation, by providing evidence on how plants coordinate photosynthetic adjustments and cycling of damaged resources under stressed conditions. As a prerequisite for elucidating this mechanism, we tried to elucidate the function of APE1 in chloroplasts. Here, we demonstrated the stromule localization of APE1 and verified its interaction with ATI1, which suggests its yet-to-be-developed function in autophagy and the transmission of signals under stressed conditions.

## Materials and methods

### Plant materials

*Arabidopsis thaliana* Col-0 wild-type (WT) seeds were obtained from the Arabidopsis Biological Resource Center (ABRC) at Ohio State University (Columbus, OH, USA). The seeds underwent surface sterilization with 75% ethanol and were stratified at 4°C in the dark for 3 d. Seeds were then germinated on Murashige and Skoog (MS) plates containing 2% sucrose and 0.8% agar, maintained at 22°C under 100 µmol photons m^−2^ s^−1^ irradiance with a 16-h light/8-h dark cycle. After 2 weeks, seedlings were transferred to a 1:1:1 mixture of peat moss, vermiculite, and perlite, where they continued to grow under identical environmental conditions.

Seeds of *Nicotiana benthamiana* were cultivated following standard growth procedures as detailed in a previous publication.^12^

### Gene expression analysis

RNA extraction was performed using RNAiso reagent (TaKaRa, Shiga, Japan), followed by reverse transcription with the PrimeScript^TM^ Double Strand cDNA Synthesis Kit (TaKaRa) according to the manufacturer’s protocols. From the resulting cDNA pool, we individually amplified the full-length open reading frames (ORFs) of *APE1* (*At5g38660*), *ATI1* (*At3g47620*), *LIL3.1* (*At4g17600*), and *RecA1* (*At1g79050*). Transcript levels were quantified via quantitative real-time PCR (qPCR) using SYBR Premix ExTaq II (TaKaRa) on a Thermal Cycler Dice Real-Time System TP800 (TaKaRa), following the manufacturer’s manual. Expression level of each gene was calculated using the comparative *C*_T_ method,^13^ with *ACTIN2* (*At3g18780*) serving as the reference gene.^14^ Each analysis included a minimum of three biological replicates, and all experiments were conducted in triplicate. The complete list of primers used in this study can be found in Supplementary Table S1.

### Antibodies

For the generation of APE1-specific antibodies, a synthetic peptide corresponding to amino acids Cys^173^–Gln^187^ was used as an antigen for rabbit immunization by GenScript (Nanjing, China). The resulting antiserum was purified via immunoblotting prior to experimental use.^15^ Commercial antibodies were sourced as follows: anti-RbcL from Sangon (Shanghai, China), while anti-LHCB1 and anti-TOC75 were obtained from Agrisera (Vännäs, Sweden). Secondary antibodies – horseradish peroxidase (HRP)-conjugated goat anti-mouse or rabbit IgGs (H+L) – were purchased from Beyotime (Shanghai, China). Detection was performed using Immobilon Western Chemiluminescent HRP Substrate (Beyotime) according to the manufacturer’s protocol, with membrane imaging conducted on a cooled charge-coupled device (CCD) system (Tanon, Shanghai, China).

### Protein–protein interaction assays

To evaluate protein interactions through yeast two-hybrid (Y2H) analysis, full-length ORFs of *ATI1* and *APE1* were inserted into pGAD-T7 and pGBK-T7 vectors (TaKaRa), respectively. Negative controls consisted of the corresponding empty vectors. *Saccharomyces cerevisiae* strain AH109 cells co-expressing both fusion proteins were cultured and then spotted onto both nonselective double drop-out plates (DDO, SD/–Leu/–Trp) and selective quadruple drop-out plates (QDO, SD/–Leu/–Trp/–His/–Ade) containing 5-bromo-4-chloro-3-indolyl-β-D-galactopyranoside (X-α-Gal). This procedure was performed according to the manufacturer’s protocol to verify protein interactions. Documentation of results was conducted 3–6 d after inoculation.

For luciferase complementation assay (LCA), the *LUC+* gene from pSP-Luc+NF (Promega, Madison, WI, USA) was divided into 1242-bp nLUC and 459-bp cLUC halves. Both halves were separately cloned into pCAMBIA1300 (CAMBIA, Canberra, Australia) between the enhanced cauliflower mosaic virus (CaMV) *35S* promoter and the Heat Shock Protein (HSP) terminator from *A. thaliana*.^16^ Full-length ORFs encoding the protein pair to be tested were separately cloned to transcriptionally fuse with nLUC and cLUC. Each of the constructs was transformed into *Agrobacterium tumefaciens* GV3101 by electroporation.^17^ A plasmid containing the *P19* silencing suppressor (kindly provided by Dr. David Baulcombe, Cambridge University, UK) was individually transformed into *Agrobacterium*. Transformed *Agrobacterium* cells were cultivated, collected, and then resuspended in the infiltration media (10 mM MgCl_2_, 200 μM acetosyringone) to an OD_600_ of 0.8. Cultures carrying the genes of interest and *P19* were equally mixed before infiltration on the abaxial leaf surface of *N. benthamiana*. The transfected plants were allowed to grow for 2–3 d under a 16-h light/8 h dark-light cycle and injected with 0.2 mg mL^−1^ luciferin (potassium salt) (Yeasen, Shanghai, China). Images were collected using a NightShade LB 985 In Vivo Plant Imaging System (Berthold, Baden-Württemberg, Germany).

### Chloroplast isolation and sub-fractionation

Chloroplast isolation was performed following the protocol previously described by Huang et al.^18^ The purified chloroplasts were subjected to lysis in TE buffer (10 mM Tris-HCl, 1 mM EDTA, pH 7.5) with a 30-min incubation on ice, followed by centrifugation at 3000 *g* for 5 min at 4°C to isolate thylakoid membranes. The resulting supernatant underwent ultracentrifugation at 288,000 *g* for 1 h on a 0.46/0.80/1.00 M sucrose density gradient to fractionate the sub-chloroplast components. We separately collected the stromal protein fraction (top layer), outer envelope membrane proteins (interface between 0.46 M and 0.8 M layers), and inner envelope membrane proteins (interface between 0.8 M and 1 M layers). Each fraction was combined with an equal volume of 2× SDS loading buffer^19^ supplemented with 1 mM DTT, 1 mM phenylmethylsulfonyl fluoride (PMSF), and 1× Protease Inhibitor Cocktail (New England Biolabs, Ipswich, MA, USA). Samples were then denatured by boiling for 5 min and centrifuged at 10,000 *g* for 10 min at 4°C. The clarified supernatants were subsequently used for protein separation by SDS-PAGE and immunoblot analysis according to standard protocols.^19^

### Microscopy

For subcellular location observation, ORFs encoding APE1, LIL3.1, and the transit peptide region (Met^1^–Ala^52^) of RecA were cloned into the *Nco* I site of pCNHP, which we constructed based on pCAMBIA1300 harboring sequentially the *35S* promoter, the synthetic 5’ and 3’ untranslated regions of Cowpea mosaic virus RNA2 flanking the coding region fused in frame to the 5’-end of the gene for either enhanced yellow fluorescent protein (EYFP) or mCherry, and the Heat Shock Protein (HSP) terminator from *A. thaliana*, as previously described.^20^ For the infiltration of *N. benthamiana* leaves, the constructs described above and the plasmid containing the *P19* silencing suppressor were individually transformed into *Agrobacterium tumefaciens* strain GV3101 by electroporation.^17^ Cultivation of *Agrobacterium* and infiltration of *N. benthamiana* were performed as described above. Fluorescent signals were observed 3–5 d post-infiltration.

An LSM 880 confocal laser scanning microscope system (Carl Zeiss, Oberkochen, Germany) was used to image fluorescent proteins. EYFP was excited at 488 nm and recorded at 500–530 nm, mCherry was excited at 543 nm and recorded at 580–620 nm, and chlorophyll autofluorescence was excited at 543 nm and recorded at 655–755 nm.^21^ Images were analyzed using ZEN3.1 software (Blue Edition, Carl Zeiss). Co-localization intensity was calculated using the same software. At least three replicates were analyzed for each sample. To aid visualization, we also plotted the intensities of different signals along defined lines.^22^

### Statistical analysis

GraphPad Prism 9 (GraphPad Software, San Diego, CA, USA) was used for statistical analysis. We employed ANOVA followed by Tukey’s test to determine statistical significance. Values with the same letters are significantly different from each other at the *p* < 0.001 level.

## Results and discussion

**Figure 1.**
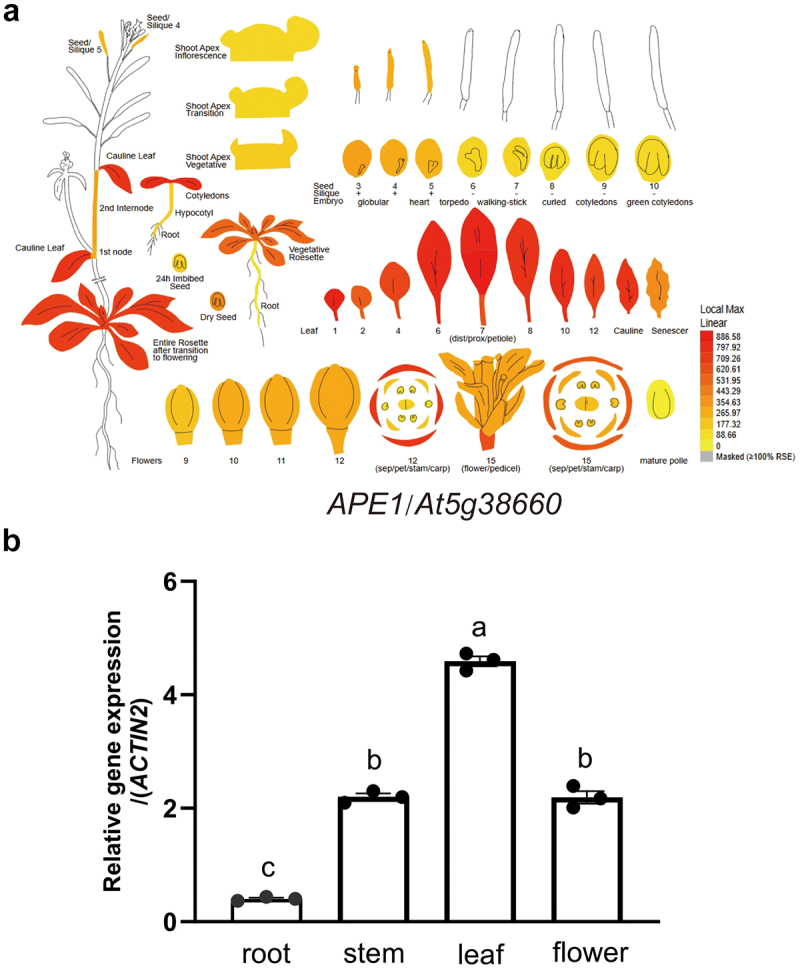
Expression pattern of *APE1*. (a) Analysis of *APE1* expression pattern from the Bio-Analytic Resource for Plant Biology. (b) Transcript abundance of *APE1* in different tissues determined by qPCR and normalized against the level of *ACTIN2*. Data are means±SE (*n*=3). Letters above histograms indicate significant differences as determined by Tukey’s HSD method (*p*<0.001).

To elucidate the function of APE1, we first checked the expression pattern of its coding gene at the Bio-Analytic Resource for Plant Biology (http://www.bar.utoronto.ca). Our search indicated a predominant expression of *APE1* in rosette leaves (Figure 1a). We then isolated total RNA from roots, stems, rosette leaves, and flowers from the wild-type (WT) *A. thaliana* seedlings, and performed reverse transcription and qPCR quantification. Our determination also confirmed that *APE1* is mainly expressed in leaves (Figure 1b).

**Figure 2.**
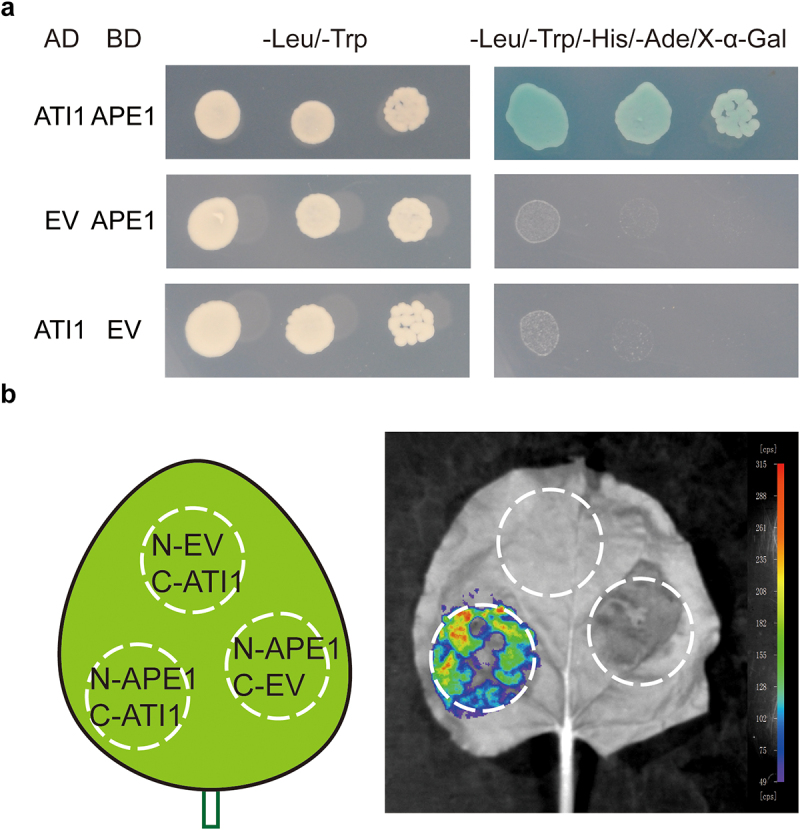
APE1 interacts with ATI1. (a) Yeast two-hybrid assay. *APE1* was cloned into pGBK-T7 with the DNA-binding domain (BD) and *ATI1* into pGAD-T7 with the activation domain (AD). Yeast AH109 cells were co-transformed with a combination of the indicated plasmids or empty vector (EV) and plated onto non-selective (DDO, –L/–W) plates and selective (QDO, –L/–W/–H/–A) plates containing X-α-Gal and 3-AT (80 mM) to suppress self-activation. (b) Luciferase complementation assay. APE1 was fused with the N-half of LUC (N-APE1), and ATI1 with the C-half of LUC (C-ATI1). *N. benthamiana* leaves were transfected to co-express different combinations of proteins as indicated. Empty vector (EV) was used as the negative control. Three days after transfection, luciferin at 0.2 mg mL^−1^ was supplied as the substrate, signals were collected, and representative image is shown. The color-coded bar displays the intensity of LUC activity.

We then tried to confirm the protein–protein interaction between APE1 and ATI1. We first performed the Y2H assay to assess the interaction *in vivo*. Yeast cells expressing ATI1 with APE1 could grow on selective QDO plates containing X-α-gal and turned colonies blue, while the negative ones with the empty vector could not, suggesting a positive interaction between these two proteins (Figure 2a). The *in planta* interaction was detected in *N. benthamiana* mesophyll cells using LCA, with APE1 fused to nLUC and ATI1 fused to cLUC. Combinations of the fusion proteins were co-expressed in *N. benthamiana* leaves. Three days after transfection, the enzymatic activity of the reconstituted LUC was detectable in the transfected regions, indicating the *in planta* interaction between APE1 and ATI1 (Figure 2b). No fluorescent signal was detectable in the control regions where either of the interacting pair was absent.

**Figure 3.**
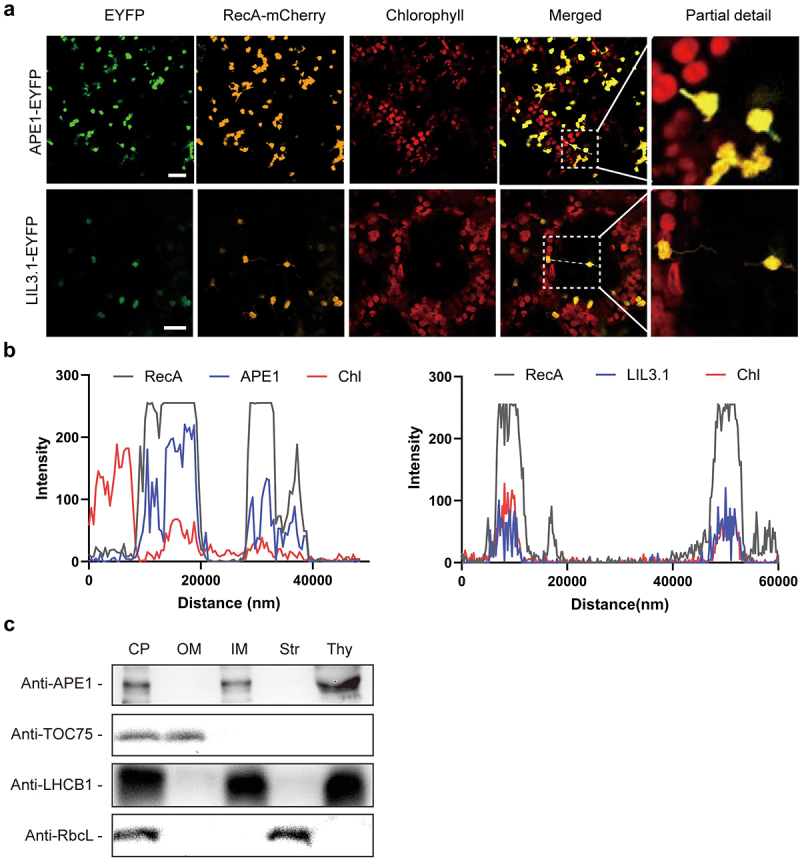
Subcellular localization of APE1. (a) Co-localization of APE1 and stromule in *N. benthamiana*. The full-length APE1 and LIL3.1 were fused with EYFP and expressed separately in *N. benthamiana* leaves. The mCherry protein fused after the transit peptide of RecA was used as a marker to indicate stromules. For each sample, representative images under EYFP, mCherry, and chlorophyll autofluorescence channels and the merged signals are shown (Bar = 20 μm). (b) Intensities of different signals along an arbitrary line in the merged image in (a) were plotted. (c) Distribution of APE1 in fractionated chloroplasts. Intact chloroplasts were isolated from *A. thaliana* WT seedlings and sub-fractionated for immunoblot analysis. TOC75, LHCB1, and RbcL were probed as markers indicating different fractions. CP, total chloroplast; OM, outer envelope membrane; IM, inner envelope membrane; Str, stroma; Thy, thylakoid membrane.

Since ATI1 was reported to have a stromule localization,^10^ we tried to determine whether APE1 is also present in stromules by overexpressing APE1 fused with an EYFP tag in *N. benthamiana* mesophyll cells. In this study, we fused the mCherry fluorescent protein after the transit peptide (Met^1^–Ala^52^) of RecA as a stromule marker.^23^ After infiltration and a 3-d growth, our confocal observation clearly indicated that APE1 localized both inside chloroplasts and in the stromules, similar to the localization of RecA (Figure 3a). As a control, we expressed the thylakoid membrane protein LIL3.1^24^ fused with the EYFP tag in parallel. However, its signal was only observed inside chloroplasts (Figure 3a). We further plotted the intensities of different signals along an arbitrary line, as indicated in the merged image.^22^ The plot clearly showed a full overlap of the APE1-EYFP signal with those of RecA-mCherry and chlorophyll autofluorescent, whereas that of LIL3-EYFP only partially overlapped with the latter one (Figure 3b).

Because APE1 was reported as a chloroplast protein,^10^ and we did observe the APE1-EYFP signal inside chloroplasts (Figure 3b), we tried to figure out its sub-organelle localization. We first isolated intact chloroplasts from WT leaves and separated them into the inner and outer envelope membranes, the stroma, and the thylakoid membrane fractions. After SDS-PAGE separation, our immunoblotting analysis indicated that APE1 predominantly accumulated in the thylakoid fraction and partially localized in the inner envelope membrane (Figure 3c), which participates in the formation of the stromules, together with the marker protein LHCB1. We did not observe the presence of APE1 in the outer envelope membrane, nor the stroma, fractions, for which TOC75 and the large subunit of Rubisco (RbcL) were used as markers, respectively (Figure 3c).

In this study, we demonstrated the localization of APE1 in stromules, and validated its interaction with ATI1. Stromules are thin tubular extensions of the plastid compartment surrounded by the envelope membrane and emanate from all types of plastids found in vascular plants and increase in frequency when exposed to reactive oxygen species, sugars, hormones, pathogen effector proteins, and in chloroplast division mutants or transformed cells overexpressing plastid outer-envelope proteins.^25^ Previous research has suggested that stromules may also play a role in recycling chloroplast proteins under suboptimal environmental conditions.^26^ Another widely accepted function of stromules is facilitating signal exchange between plastids and other subcellular compartments, especially under stressed conditions. A study on the application of H_2_O_2_ or SA to tobacco (*N. tabacum*) leaves reported the transfer of signaling proteins and metabolites, such as H_2_O_2_, to the nucleus directly from the chloroplast with the help of stromules.^27^ Since previous reports suggested the contribution of APE1 to the acclimation of photosynthetic system to stressed conditions,^11^ we speculate it might function through either communicating signals and/or recycling proteins from the damaged chloroplasts to the surrounding environment, the latter of which is probably mediated by ATI1. Further studies on their dynamic interaction might help to better understand how autophagy is involved in the response of chloroplasts to challenging conditions.

## Supplementary Material

Table S1.docx
