# Supplementary material for: APE1 localizes to chloroplast stromules and interacts with ATI1 in Arabidopsis
Source: Plant Signal Behav. 2025 May 30;20(1):2511830. doi: 10.1080/15592324.2025.2511830 (PMC12128654; doi:10.1080/15592324.2025.2511830)
Supplement: Table S1.docx [file KPSB_A_2511830_SM3462.docx]

**Supplemental Table S1.** Primers used in this study.

| Primer name | Primer sequence (5’-3’)1 |
| --- | --- |
| For gene cloning | |
| APE1-ORF-F | ATGGGATCTATAACGGTAGCT |
| APE1-ORF-R | AGAGGTAGAAGATACAGAGTTGG |
| ATI1-ORF-F | ATGGCTAACAATGAGGAGCAT |
| ATI1-ORF-R | GACCTCACTGGAGGAGCC |
| LIL3.1-ORF-F | ATGGCGTTGTTCTCCCCG |
| LIL3.1-ORF-R | GATGAGTCATTGGGTTCTAAGAAGAAG |
| RecA-F | ATGGATTCACAGCTAGTCTTGTCT |
| RecA-R | ATCGAATTCAGAACTGATTTTGTGGG |
| For subcellular localization | |
| APE1-pCNHP-EYFP-F | attctgcccaaattcgcgccATGGGATCTATAACGGTAGCTCCGG |
| APE1-pCNHP-EYFP-R | tcctcgcccttgctcaccatAGAGGTAGAAGATACAGAGTTGGTGGA |
| LIL3.1-pCNHP-EYFP-F | ctgcccaaattcgcgccatATGGCGTTGTTCTCCCCG |
| LIL3.1-pCNHP-EYFP-R | gagctgcacgctgccAGAGGTAGAAGATACAGAGTTGGTGGA |
| RecA-pCNHP-mCherry-F | attctgcccaaattcgcgACTAGTATGGATTCACAGCTAGTC |
| RecA-pCNHP-mCherry-R | ctcgcccttgctcaccatGTCTCTATCGAATTCAGA |
| For luciferase complementation assay | |
| APE1-pCAMBIA1300-nLUC-F | ccatttacgaacgatagccATGGGATCTATAACGGTAGCT |
| APE1-pCAMBIA1300-nLUC-R | tttttggcgtcggtgaGAGGTAGAAGATA |
| ATI1-pCAMBIA1300-cLUC-F | tcaccatttacgaacgatagcCATGGCTAACAATGAGGAGCAT |
| ATI1-pCAMBIA1300-cLUC-R | gtttacataaccggCCATGACCTCACTGGAGGAGCC |
| For qPCR | |
| APE1-QPCR-F | ATATGGGCTGGGAGGAGGAA |
| APE1-QPCR-R | TTTACCGACTTCTGCCGTGA |
| ACTIN2-QPCR-F | TTGACTACGAGCAGGAGATGG |
| ACTIN2-QPCR-R | ACAAACGAGGGCTGGAACAAG |

^1^ Nucleotides in lowercase are designed for cloning through homologous recombination.
